# Outcome of Pediatric Patients With Sickle Cell Anemia Admitted With Fever: A Retrospective Single-Center Study

**DOI:** 10.7759/cureus.69570

**Published:** 2024-09-17

**Authors:** Mohammed A Zolaly, Abeer Alharbi, Suhaylah Algrafi, Maryam Balkhair, Jumanah Aljohani, Marwah M Quordi, Lujain Alharbi, Waheed A Turkistani

**Affiliations:** 1 Hematology and Oncology, Taibah University, Medina, SAU; 2 Medicine, Taibah University, Medina, SAU; 3 Pediatrics, King Salman Medical City, Ministry of Health, Medina, SAU

**Keywords:** complications, fever, outcomes, saudi arabia, sickle cell anaemia

## Abstract

Background: Pediatric patients with sickle cell anemia (SCA) are known to have an increased susceptibility to infections, leading to a higher incidence of fever among this population. However, there is limited literature specifically focusing on the outcomes of pediatric SCA patients presenting to the emergency department (ED) or hospital with a primary diagnosis of fever.

Objectives: The objective of this retrospective single-center study was to compare the characteristics of fever and associated symptoms among pediatric patients with SCA, and to investigate the risk factors associated with patients' outcomes and mortality in this specific population.

Patients and methods: The study was conducted at the King Salman Medical City, Maternity and Children's Hospital, Medina, Saudi Arabia, during the period from 2017 to 2022. All pediatric SCA patients under the age of 14, who presented to the ED of the hospital during the study period with a primary diagnosis of fever, were included. The study collected and analyzed clinical, laboratory, treatment, complications, and outcome data of the patients using appropriate statistical methods, including logistic regression.

Results: A total of 57 children were included in the analysis. The mean age of the patients was 7.1 ± 4.3 years, with 26 males (45.6%) and 31 females (54.4%). Among the cases, 8 (14%) exhibited fever along with gastrointestinal symptoms, 18 (31.6%) presented with musculoskeletal symptoms, 5 (8.8%) showed neurological symptoms, and approximately half of the cases (45.6%) displayed respiratory symptoms along with fever. Logistic regression analysis identified several significant factors associated with complications in this sample, including a very low level of hemoglobin (Hb) (<7 g/dL) with an odds ratio (OR) of 14.5 (95% CI=1.03-222.3), ICU admission with OR of 14 (95% CI=1.03-186.8), and a hospital stay duration of more than 10 days (OR=11.5; 95% CI=1.10-121.3). Additionally, fever associated with neurological symptoms, neutrophilia, history of splenectomy, and male sex showed positive associations with complications among the studied patients, although not significant.

Conclusion: This study provides valuable insights into the characteristics and outcomes of febrile pediatric patients with SCA. The findings highlight the importance of early recognition and management of fever in this vulnerable population, particularly when certain risk factors are present.

## Introduction

Sickle cell disease (SCD) is an autosomal recessive genetic disorder caused by a single-point mutation in the β-chain of the hemoglobin (Hb) gene that results in the replacement of glutamic acid with valine in the Hb protein, resulting in hemoglobin S (HbS) formation [1]. HbS is vulnerable to multiple stressful events that lead to deoxygenation and polymerization reactions, resulting in sickling of the red blood cells (RBCs) [2]. RBCs with sickle-like morphology become more rigid and “sticky” compared to normal RBCs, causing frequent vaso-occlusive episodes and depriving tissues and organs of oxygen [3].This process predisposes sickle cell patients to a variety of acute and chronic complications early in their lives.

Sickle cell patients mostly present to the pediatric emergency department (ED) with fever caused by viral or bacterial infection, or sickle cell crisis [4]. The underlying causes for these complications result from vascular occlusion, increased blood viscosity, leading to functional asplenia, and immune deficiency. This pathology leads to life-long increased susceptibility to serious bacterial infections [5]. This is proven by some studies which show the incidence of bacterial infection in children with SCD globally is 16% compared to 3-14% in general children [6]. Because of the high morbidity and mortality rate associated with bacterial infection, any patient presented to the pediatric ED with a febrile episode will require an immediate evaluation, including blood cultures and initiation of empiric antibiotics [7]. There are a few studies that measure the outcome of fever in sickle cell patients one of them was done in Jeddah and it reported the rate of bacterial infection among pediatric patients presenting to the ED with fever to be 8.6% [8]. Another study in the USA done over a period of 10 years found the rate of bacterial infection was 16% in febrile children with SCD [6].

Genetic disorders such as SCD are very common in Saudi Arabia. Unfortunately, although the prevalence of SCD has been decreasing in every region of Saudi Arabia, the prevalence remains higher than that in other countries [9]. In 2015, a high prevalence rate of 49.6% (45.8% for the trait and 3.8% for the disease) was reported in Saudi Arabia [10] .This could be referred to the high percentage of consanguineous marriages in the country, which present a great obstacle in controlling SCD in Saudi Arabia [2]. Considering the burden and prevalence of this disease, the Saudi government in 2004 directed its efforts toward the primary prevention of this disease through mandatory premarital screening and genetic counselling program (PMSGC) for all couples planning to marry. Couples who are at risk were offered genetic counselling. As a result, a five-fold increase in voluntary cancellation was observed among at-risk couples from 2004 to 2009 [11]. Currently, there is a lack of data on the outcomes of febrile events in pediatric SCD patients in Medina. Consequently, we conducted this study to evaluate and assess their outcomes.

## Materials and methods

This retrospective study follows a cross-sectional design and focuses on pediatric patients with sickle cell anemia (SCA) who were admitted to the King Salman Medical City, Maternity and Children's Hospital, Medina, Saudi Arabia, during the period from 2017 to 2022, with a primary diagnosis of fever. The study included all pediatric patients under the age of 14 years diagnosed with SCA and presented to the ED with a fever of 38°C and more during the study period. Fever was grouped into the following categories according to associated symptoms: fever with gastrointestinal symptoms, fever with musculoskeletal symptoms, fever with neurological symptoms, and fever with respiratory symptoms.

Data extraction

Relevant data were extracted from the electronic medical records system. The following information was included: Demographic characteristics: age, gender, and nationality. Clinical presentation included symptoms, clinical signs, heart rate, respiratory rate, and any associated symptoms. Laboratory findings included leukocyte count, neutrophils count, erythrocyte sedimentation rate (ESR), Hb level, blood cultures, and other relevant laboratory tests. Hb level was classified as “low” (<10 g/dL), and “very low” (<7.9 g/dL). Treatment and management included antibiotic therapy, supportive care, and a history of blood transfusion and splenectomy. Length of hospital stay. The outcome data included the resolution of fever, complications during hospitalization, need for intensive care, and mortality. All outcome data were defined as a binary variable (Yes vs No). All predictors were collected prior to the occurrence of the complication at or during the time of hospital admission, ensuring that the temporal relationship between the predictors and the outcome is preserved.

Ethical considerations

This study followed ethical guidelines and regulations applicable to retrospective studies. The research proposal obtained approval from both the King Salman bin Abdelaziz Medical City Institutional Review Board (IRB log No: 023-026) and the Research Ethics Committee of Taibah University (IORG0008716-IRB00010413). The study prioritized the protection of data privacy and confidentiality, strictly utilizing the data for research purposes only.

Statistical analysis

Statistical analyses were performed using SPSS 22.0 for Windows (Chicago, USA). Data were presented using frequencies for categorical data and means and standard deviation (SD) for continuous data. The incidence of fever among the studied cases was tabulated and then compared by the studied patients' characteristics using chi-square, fisher exact, T-test, and ANOVA analysis as appropriate. The complication was also compared to the studied patients’ characteristics. Stepwise logistic regression analysis was also done (with an inclusion criterion of 10% and an exclusion criterion of 20%), to predict the most important factors associated with complications. The statistical significance level was set at p<0.05.

## Results

This study enrolled a total of 57 children who were diagnosed with SCA. The average age of the participants was 7.1 ± 4.3 years, and 45.6% of them were younger than seven years old. Among the participants, 45.6% were male (n=2). The study findings revealed that tachycardia was present in 71.9% of the patients, while tachypnea was observed in 52.6% of them. Laboratory investigations indicated that 61.4% of the patients had low Hb levels, and 12.3% had very low Hb levels. Leukocytosis was found in 45.6% of the patients, neutrophilic in 14%, and high ESR in 43.9%. No cases showed positive blood culture in the sample. 

Additionally, 28.1% of the participants had a history of splenectomy, 63.2% had received blood transfusions, 43.1% had received ospen prophylaxis, and 48.3% had received influenza vaccination. The mean duration of hospital stay was 7.4 ± 6.8 days, with four cases (7%) requiring admission to the ICU. Complications were observed in only four cases (7.1%); three cases with organ failure and one case of death (Table 1).

**Table 1:** Characteristics of the studied 57 patients * Data are presented by mean ± SD or by n (%). Hb: Hemoglobin; ESR: Erythrocyte sedimentation rate; HU: Hydroxyurea

Characteristics*	N=57
Age in years; mean ± SD (range)	7.1 ± 4.3 (1-14 years)
Age in years	
< 7	26 (45.6)
≥ 7	31 (54.4)
Gender	
Male	26 (45.6)
Female	31 (54.4)
Nationality	
Saudi	35 (61.4)
Non-Saudi	22 (38.6)
Tachycardia	41 (71.9)
Tachypnea	30 (52.6)
Level of Hb	
Normal	15 (26.3)
Low (<10 g/dL)	35 (61.4)
Very low (<7.9 g/dL)	7 (12.3)
Leukocytosis	26 (45.6)
Neutrophilia	8 (14.0)
ESR (mean ± SD)	25 (43.9)
Positive blood culture	0 (0.0)
History of splenectomy	16 (28.1)
History of blood transfusion	36 (63.2)
History of outpatient follow-up	40 (70.2)
Ospen prophylaxis	25 (43.1)
Influenza vaccination	28 (48.3)
HU usage	44 (75.9)
Folic acid usage	43 (75.4)
Length of hospital stay (mean ± SD)	7.4 ± 6.8
ICU admission	4 (7.0)
Complications	
No	53 (93.0)
Organ failure	3 (5.3)
Death	1 (1.8)

**Figure 1:**
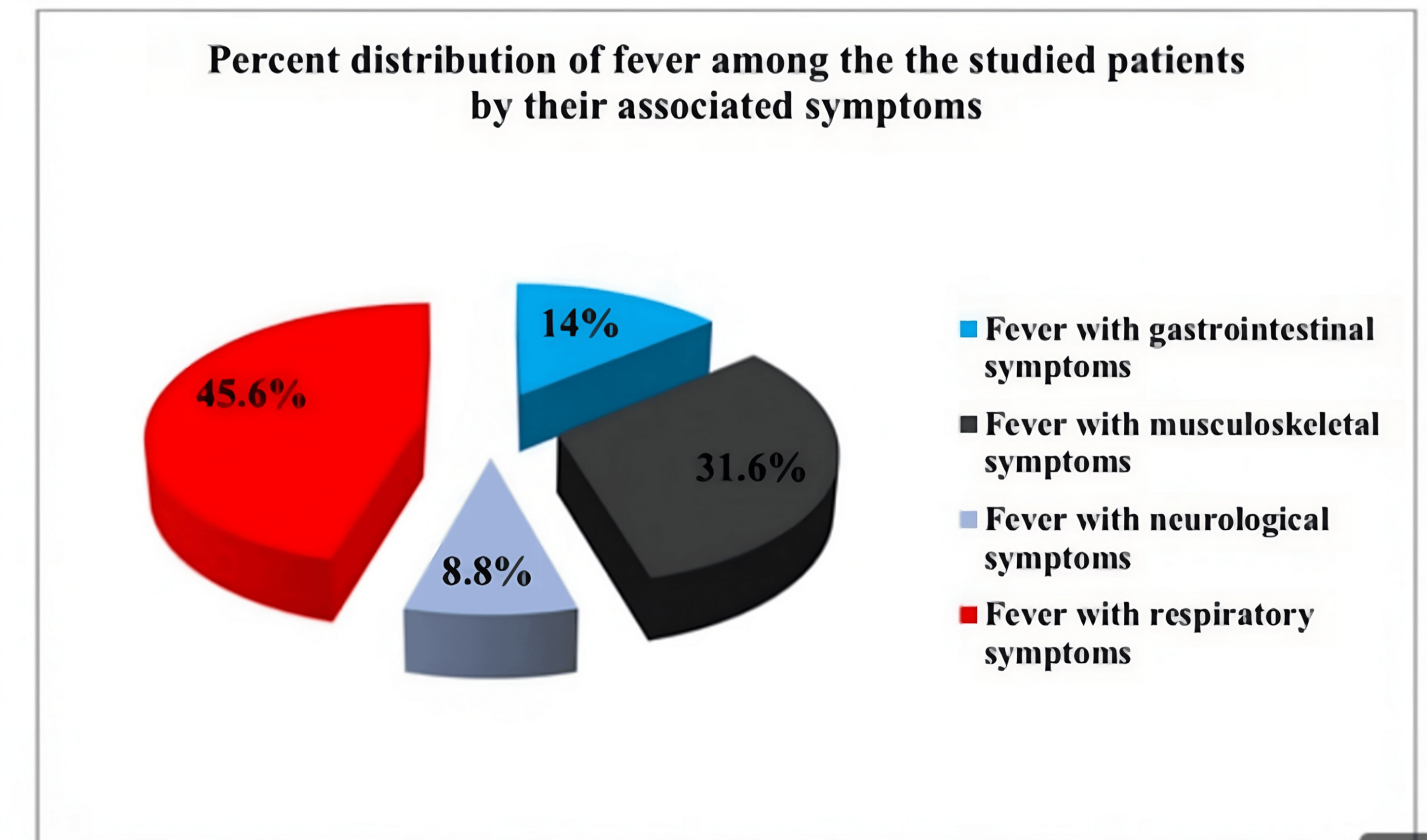
Percent distribution of fever among the the studied patients by their associated symptoms

Table 2 presents the different manifestations of fever observed in the patients included in the study. Among the cases, 8 instances (14%) exhibited fever along with gastrointestinal symptoms, 18 cases (31.6%) presented fever along with musculoskeletal symptoms, 5 cases (8.8%) showed fever along with neurological symptoms, and approximately half of the cases (45.6%) displayed fever along with respiratory symptoms. The percent distribution of fever by associated symptoms is depicted in Figure 1.

**Table 2:** Fever presentation among the studied 57 patients

Fever presentation	N	%
Fever with gastrointestinal symptoms	8	14.0
Fever with musculoskeletal symptoms	18	31.6
Fever with neurological symptoms	5	8.8
Fever with respiratory symptoms	26	45.6

Table 3 shows the comparison of fever groups based on clinical and biological variables in the patients under study. Statistically significant differences were observed in various aspects, including the nationality of the patients, Hb levels, influenza vaccination, hydroxyurea (HU) usage, and the incidence of complications. However, no statistically significant differences were found for other variables examined. Fever accompanied by gastrointestinal symptoms was more prevalent in younger children, with an average age of 4.9 ± 2.9 years. It was also more common in patients who received influenza vaccination (75%), had a history of folic acid usage (100%), had a history of blood transfusion (75%), and had regular outpatient follow-up (87.5%).

**Table 3:** Clinical and biological variables in the studied patients by their groups of fever Group 1: Fever with gastrointestinal symptoms; Group 2: Fever with musculoskeletal symptoms; Group 3: Fever with neurological symptoms: Group 4: Fever with respiratory symptoms Low Hb: <10 g/dL; Very low Hb: <7.9 g/dL **Except for age in years and length of hospital stays (one-way ANOVA analysis), all other variables were compared using the Fisher exact test. ***Significant Hb: Hemoglobin; ESR: Erythrocyte sedimentation rate; HU: Hydroxyurea

Variable	Group 1*	Group 2*	Group 3*	Group 4*	Test value**	P value
	(n= 8)	(n= 18)	(n= 5)	(n= 26)		
Age in years	4.9 ± 2.9	7.5 ± 5.1	6.2 ± 4.1	7.7 ± 4.1	1.1	0.41
Male sex	2 (25.0)	6 (44.4)	4 (80.0)	12 (46.2)	3.53	0.32
Saudi nationality	2 (25.0)	3 (72.2)	2 (40.0)	18 (69.2)	7	0.04***
Tachycardia	7 (87.5)	14 (77.8)	4 (80.0)	16 (61.5)	2.47	0.49
Tachypnea	5 (62.5)	7 (38.9)	4 (80.0)	14 (53.8)	3.1	0.39
Low Hb	1 (12.5)	11 (61.1)	3 (60.0)	20 (76.9)	16.1	0.01***
Very low Hb	3 (37.5)	0 (0.0)	1 (20.0)	3 (11.5)		
Leukocytosis	1 (12.5)	10 (55.6)	2 (40.0)	13 (50.0)	4.5	0.2
Neutrophilia	1 (12.5)	3 (16.7)	0 (0.0)	4 (15.4)	0.7	0.86
High ESR	2 (25.0)	8 (44.4)	2 (40.0)	13 (50.0)	1.6	0.68
History of splenectomy	3 (37.5)	5 (27.8)	2 (40.0)	6 (23.1)	1.4	0.73
History of blood transfusion	6 (75.0)	10 (55.6)	4 (80.0)	16 (61.5)	1.5	0.71
Outpatient follow up	7 (87.5)	14 (77.8)	3 (60.0)	16 (61.5)	2.8	0.44
Ospen prophylaxis	3 (27.5)	9 (50.0)	0 (0.0)	13 (50.0)	4.6	0.2
Influenza vaccination	6 (75.0)	11 (61.1)	3 (60.0)	8 (30.8)	7.6	0.04***
HU usage	4 (50.0)	18 (100.0)	3 (60.0)	19 (73.1)	11.1	0.01***
Folic acid usage	8 (100.0)	13 (72.2)	2 (40.0)	20 (79.9)	3.8	0.1
Length of hospital stay	6.6 ± 4.1	6.8 ± 7.1	7.6 ± 6.3	8.1 ± 6.8	0.45	0.94
ICU admission	0 (0.0)	1 (5.6)	1 (20.0)	2 (7.7)	2.1	0.55
Complications					9.6	
Death	0 (0.0)	0 (0.0)	1 (20.0)	0(0.0)		
Organ failure	1 (12.5)	2 (11.1)	0 (0.0)	0 (0.0)		0.04***

Fever associated with neurological symptoms was more prevalent among male patients (80%) and those with low Hb levels. Fever accompanied by respiratory symptoms was more prevalent in older children, with an average age of 7.7 ± 4.1 years. It was also more common in patients with low Hb levels (76.9%), a history of blood transfusion, regular outpatient follow-up (61.5%), folic acid usage (79.9%), and HU usage (73.1%). Patients with fever accompanied by respiratory symptoms had a longer mean length of hospital stay, while those with fever associated with neurological symptoms had a higher rate of ICU admission. Among the cases, three patients experienced organ failure (one in the fever with gastrointestinal symptoms group and two in the fever with musculoskeletal symptoms group), and one patient from the fever with neurological symptoms group unfortunately passed away.

Table 4 displays the clinical and biological variables in the patients under study categorized by the presence of complications. Statistically significant differences were found between patients with complications and those without complications in terms of their Hb levels, history of blood transfusion, receiving ospen prophylaxis, ICU admission, and length of hospital stay. However, no statistically significant differences were observed for other variables examined.

**Table 4:** Clinical and biological variables in the studied patients by complications Low Hb: <10 g/dL; Very low Hb: <7.9 g/dL *Significant ** Except for age in years and length of hospital stays (independent t test), all other variables were compared using the Fisher exact test. Hb: Hemoglobin; ESR: Erythrocyte sedimentation rate; HU: Hydroxyurea

Variable	Complications	No complications	Test value**	P value
	(n= 4)	(n= 53)		
Age in years	5.5 ± 4.7	7.1 ± 4.3	0.75	0.45
Male sex	3 (75.0)	23 (43.4)	0.49	0.32
Saudi nationality	2 (50.0)	33 (62.3)	0.23	0.65
Fever presentation				
With gastrointestinal symptoms	1 (25.0)	7 (13.2)		
With musculoskeletal symptoms	2 (50.0)	16 (30.2)		
With neurological symptoms	1 (25.0)	4 (7.5)		
With respiratory symptoms	0 (0.0)	26 (49.1)	5.2	0.1
Tachycardia	2 (50.0)	28 (52.8)	0.3	0.81
Tachypnea	3 (75.0)	38 (71.7)	0.02	0.88
Low Hb	0 (0.0)	35 (66.0)	7.9	
Very low Hb	2 (50.0)	5 (9.4)		0.01*
Leukocytosis	2 (50.0)	24 (45.3	0.03	0.85
Neutrophilia	1 (25.0)	7 (13.2)	0.5	0.55
High ESR	2 (50.0)	23 943.4)	0.15	0.79
History of splenectomy	2 (50.0)	14 (26.4)	1.1	0.31
History of blood transfusion	4 (100.0)	32 (60.4)	2.5	0.28
Outpatient follow up	1 (25.0)	38 (71.7)	0.82	0.57
Ospen prophylaxis	0 (0.0)	25 (47.2)	5.8	0.03*
Influenza vaccination	2 (50.0)	26 (49.1)	0.02	0.97
HU usage	2 (50.0)	40 (75.5)	1.2	0.56
Folic acid usage	2 (50.0)	39 (73.6)	1.3	0.55
Length of hospital stay	15.0 ± 10.8	6.8 ± 6.2	-2.4	0.02*
ICU admission	2 (50.0)	2 (3.8)	6.5	0.01*
Antibiotic usage				
No	1 (25.0)	8 (15.1)		
Ceftriaxone	1 (25.0)	29 (54.7)		
Other antibiotic	2 (50.0)	16 930.2)	1.9	0.46

The logistic regression analysis revealed several factors that were examined as predictors of complications. Although not statistically significant, fever associated with neurological symptoms, neutrophilia, history of splenectomy, and male sex showed positive associations with complications, with odds ratio (OR) of 1.80, 2.50, 10.8, and 3.90, respectively. Among the predictors, the most significant factors associated with complications in this sample were a very low level of Hb (<7 g/dL), which had an OR of 14.5 (95% CI=1.03-222.3), ICU admission with an OR of 14 (95% CI=1.03-186.8), and duration of hospital stay>10 days (OR=11.5; 95% CI=1.10-121.3). Although not significant, a history of outpatient follow-up was associated with a 90% reduction in the risk of complications (OR=0.10; 95% CI=0.39-40.5), and the use of ceftriaxone antibiotic was associated with a 73% reduction in the risk of complications (Table 5).

**Table 5:** Predictors of complications among the studied cases—the results of stepwise logistic regression analysis *Significant Hb: Hemoglobin

Factors	OR	95% CI	P value
Fever associated neurological symptoms	1.80	0.10-36.5	0.71
Very low Hb (<7 g/dL)	14.5	1.03-222.3	0.04*
ICU admission	14.0	1.03-186.8	0.04*
Duration of hospital stay>10 days	11.5	1.10-121.3	0.04*
History of splenectomy	10.8	0.61-191.1	0.10
Male sex	3.90	0.39-40.5	0.24
Neutrophilia	2.50	0.15-58.2	0.56
Outpatient follow up	0.10	0.03-1.90	0.11
Ceftriaxone antibiotic	0.27	0.05-3.28	0.30

## Discussion

SCA represents a major global healthcare burden and contributes significantly to patient morbidity and mortality. This study aimed to compare the characteristics of fever and associated symptoms among pediatric patients with SCA, and to investigate the risk factors associated with patients' outcomes and mortality. The study included 57 children under the age of 14 years. No cases showed positive blood culture the studied sample. Similarly, the rate of bacteremia was also low and ranged from 1.2% to 1.5% in previously published similar studies [12,13]. Inconsistent with these results, however, was the rate of positive culture and bacteremia among febrile children with SCA: it was as high as 2.6% [7]. The rate was found to range from 1.3% to 3.6% in the earlier studies, higher than other recent publications [7,14,15]. All outcome data were defined as a binary variable (Yes vs No). All predictors were collected prior to the occurrence of the complication at or during the time of hospital admission, ensuring that the temporal relationship between the predictors and the outcome is preserved. The higher prevalence of bacteremia in these earlier studies may be influenced by factors such as regional differences in healthcare delivery, variations in clinical practice guidelines, and patient population characteristics, including age, comorbidities, and access to healthcare resources. Furthermore, the time of culture could be attributed to these observed variations. Two previous studies have identified that the mean time to a positive blood culture in SCA febrile patients was 17-23 hours and 96% of the cases would be identified within 48 hours [7,16]. Moreover, it is worth noting that temperature alone was not a reliable indicator for identifying bacteremia cases, as only a small percentage of the cases had temperatures exceeding 40°C or 39.5°C. This highlights the challenges in developing admission criteria for patients with SCA based solely on fever [7]. 

The study findings revealed that gastrointestinal symptoms were reported in 14% of the cases presenting with fever. These symptoms may include abdominal pain, nausea, vomiting, or diarrhea. The presence of gastrointestinal symptoms alongside fever suggested the possibility of an underlying infection involving the gastrointestinal tract, such as gastroenteritis or abdominal infections.Patients with SCA are found to present with a wide variety of gastrointestinal disorders mimicking vaso-occlusive episodes causing diagnostic confusion, and most of these patients present with fever [17]. 

Musculoskeletal symptoms were observed in 36.4% of the cases who presenting with fever. Musculoskeletal manifestations of fever in patients with SCA can be indicative of acute bone or joint infections, which can have serious consequences if not promptly diagnosed and treated. In a study on 85 children with SCA, 31 patients had musculoskeletal complications, giving a prevalence rate of 36.4%. The most common musculoskeletal complication was vascular necrosis, followed by septic arthritis and chronic osteomyelitis [18]. The increased susceptibility of SCD patients to musculoskeletal manifestations has long been recognized, with several mechanisms postulated, including chronic sequelae of the anemia and vaso-occlusive processes involving the musculoskeletal system [19].

Neurological symptoms were reported in 8.8% of the cases presenting with fever. The presence of neurological symptoms alongside fever raises concerns about potential complications such as central nervous system infections or cerebrovascular events in patients with SCA. Neurological complications have been reported in a previous study, and that study recommended increasing recognition of neurological complications among SCA patients to improve diagnosis and treatment options [20]. Respiratory symptoms were observed in approximately half (45.6%) of the cases with fever. Respiratory manifestations of fever in patients with SCA can be indicative of respiratory tract infections, including pneumonia or acute chest syndrome, which is a serious complication in these patients. The most common single specific pulmonary complication of SCD at all ages is acute pulmonary involvement compatible with bacterial pneumonia evidenced clinically by cough, fever, leukocytosis, pleuritic pain, and occasional dyspnea [21].

The diverse range of manifestations observed in this study highlights the complexity of febrile episodes in pediatric patients with SCA. It emphasizes the importance of a thorough clinical evaluation to identify the underlying cause of fever and associated symptoms to provide appropriate and timely interventions. Prompt recognition and management of specific manifestations, such as gastrointestinal, musculoskeletal, neurological, or respiratory symptoms, are crucial for optimizing patient outcomes and preventing complications in this vulnerable population.

The study findings revealed that complications were more prevalent among younger and male patients and in cases of fever associated with musculoskeletal symptoms. Among the antibiotics used, ceftriaxone was associated with 25% of the complications, while the other used antibiotics showed a 50% association, although this difference was not statistically significant. Ceftriaxone and cefotaxime have been recommended for the treatment of septic episodes in SCA associated with *S. pneumoniae*, Haemophilus, and Salmonella spp. infection with Yersinia enterocolitica may be treated with cefotaxime [22]. Also, the use of HU was associated with a decrease in complications. HU is a very essential drug for the care of SCA patients. Almost all major society guidelines recommend HU, and the label is extended to cover a wide range of indications in both children and adults. Long-term follow-up has shown safety, efficacy, decreased morbidity, and improved survival [23].

The significant factor associated with complications in patients with SCA in this study was an extremely low level of Hb (<7 g/dL). Patients with SCA who had severely low Hb levels had 14.5 times higher odds of experiencing complications compared to those with higher Hb levels. This finding underscores the importance of monitoring and managing Hb levels in SCA patients, as low Hb levels can contribute to disease severity and increase the risk of complications. Because sickled cells are short-lived or destroyed, there are fewer RBCs available in the body. This results in anemia. Severe anemia can make patients more susceptible to infections and complications [24].

In the present study, ICU admission was identified as a significant predictor of complications. Patients who required ICU admission had a 14 times higher odds of experiencing complications compared to those who did not require ICU care. This emphasizes the severity of illness in patients with SCA who necessitate intensive care and suggests that close monitoring and specialized interventions are crucial in managing these cases. Several previous studies have reported concurring findings of increased risk of complications and mortality among SCA patients admitted to the ICU. In addition, five-year mortality is linked with a history of long ICU stay [25,26]. In the present study, a longer duration of hospital stay (> 10 days) was also found to be significantly associated with complications. Patients with SCA who had extended hospital stays had an 11.5 times higher odds of experiencing complications. Prolonged hospitalization may be an indicator of more severe disease, increased susceptibility to infections, or the presence of other complications requiring ongoing medical management [27]. 

Although not statistically significant, the study observed that a history of outpatient follow-up was associated with a 90% reduction in the risk of complications. In a previous case-control study on 30 cases (re-admitted) and 70 controls (non-re-admitted). The greatest risk factor for readmission was no outpatient hematology follow-up in within 30 days of discharge with an OR of 7.7 (95% CI=2.4-24) [28]. This finding suggests that regular and consistent follow-up care may contribute to better disease management and early detection of potential complications, leading to improved patient outcomes. Additionally, the use of ceftriaxone antibiotic was associated with a 73% reduction in the risk of complications. This suggests that appropriate antibiotic therapy, such as ceftriaxone, may play a role in preventing or managing infectious complications in patients with SCA.

Although not significant, neutrophilia, history of splenectomy, and male sex showed positive associations with complications among the studied patients. In a previous study increased white blood cells was associated with complications of SCA patients with fever [7]. Neutrophilia is often seen in response to infection or inflammation, and an exaggerated neutrophil response may indicate a more severe inflammatory process in SCA patients with fever. Also, the absence of the spleen compromises the immune system's ability to fight infections, making splenectomized individuals more susceptible to certain bacterial infections, and there is growing concern about overwhelming post-splenectomy infection caused by encapsulated bacteria. [29]. Finally, the finding that male sex is associated with complications among SCA patients with fever suggests that male patients may be at higher risk compared to females. However, it is important to note that the reasons for this association may be multifactorial and not solely due to sex itself. Other factors, such as hormonal differences, genetic factors, or varying healthcare-seeking behaviour, could contribute to this association.

Furthermore, the lack of statistical significance in these associations may be attributed to the relatively small sample size or inherent variability in the population studied, and future research with larger sample sizes or alternative methodologies may provide clearer insights into the relationship between these variables and the occurrence of complications.

The strengths of the study include being the first to investigate this topic at the King Salman Medical City, Maternity and Children's Hospital, Medina, Saudi Arabia, based on the available literature. Moreover, the current study evaluated SCA patients with fever by the associated organ symptoms group and studied several risk factors in association with complications among these patients. Additionally, this study will provide valuable insights into the outcomes of pediatric patients with SCA admitted with fever, contributing to the understanding of managing this particular population and potentially guiding future research and clinical practices.

However, there are also some limitations to the study that should be considered. The retrospective nature of the present study may introduce inherent biases and limitations in data collection. The findings may be limited to a specific center and may not be generalizable to other settings or populations. Missing or incomplete medical records may affect the availability of certain data points. The very wide confidence intervals observed in this analysis were the result of the small sample size and the small number of cases included in the studied cells. Therefore, extensive longitudinal studies would be more suitable for further exploring the dynamic nature of these associations over time and assessing causality. 

## Conclusions

This study provides valuable insights into the characteristics and outcomes of febrile pediatric patients with SCA. The findings emphasize the importance of early recognition and management of fever in this vulnerable population, particularly in the presence of certain risk factors. They highlight the clinical factors that significantly influence complications in patients with SCA. Further research and larger-scale studies are needed to validate these findings and explore additional factors that may contribute to complications in this patient population.
